# *Hizikia fusiforme* functional oil (HFFO) prevents neuroinflammation and memory deficits evoked by lipopolysaccharide/aluminum trichloride in zebrafish

**DOI:** 10.3389/fnagi.2022.941994

**Published:** 2022-09-09

**Authors:** Ying-Ying Nie, Long-Jian Zhou, Yan-Mei Li, Wen-Cong Yang, Ya-Yue Liu, Zhi-You Yang, Xiao-Xiang Ma, Yong-Ping Zhang, Peng-Zhi Hong, Yi Zhang

**Affiliations:** ^1^Guangdong Provincial Key Laboratory of Aquatic Product Processing and Safety, Guangdong Provincial Engineering Laboratory for Marine Biological Products, Guangdong Provincial Engineering Technology Research Center of Seafood, Key Laboratory of Advanced Processing of Aquatic Product of Guangdong Higher Education Institution, Shenzhen Institute of Guangdong Ocean University, Zhanjiang Municipal Key Laboratory of Marine Drugs and Nutrition for Brain Health, Research Institute for Marine Drugs and Nutrition, College of Food Science and Technology, Guangdong Ocean University, Zhanjian, China; ^2^Collaborative Innovation Center of Seafood Deep Processing, Dalian Polytechnic University, Dalian, China

**Keywords:** oxidative stress, neuroinflammation, HFFO, memory impairment, ADMET

## Abstract

**Background:**

Oxidative stress, cholinergic deficiency, and neuroinflammation are hallmarks of most neurodegenerative disorders (NDs). Lipids play an important role in brain development and proper functioning. Marine-derived lipids have shown good memory-improving potentials, especially those from fish and microalgae. The cultivated macroalga *Hizikia fusiforme* is healthy food and shows benefits to memory, but the study is rare on the brain healthy value of its oil. Previously, we had reported that the *Hizikia fusiforme* functional oil (HFFO) contains arachidonic acid, 11,14,17-eicosatrienoic acid, phytol, and other molecules displaying *in vitro* acetylcholinesterase inhibitory and nitroxide scavenging activity; however, the *in vivo* effect remains unclear. In this study, we further investigated its potential effects against lipopolysaccharides (LPS)- or aluminum trichloride (AlCl_3_)-induced memory deficiency in zebrafish and its drug-related properties *in silica*.

**Methods:**

We established memory deficit models in zebrafish by intraperitoneal (i.p.) injection of lipopolysaccharides (LPS) (75 ng) or aluminum trichloride (AlCl_3_) (21 μg), and assessed their behaviors in the T-maze test. The interleukin-1β (IL-1β), tumor necrosis factor-α (TNF-α), acetylcholine (ACh), and malondialdehyde (MDA) levels were measured 24 h after the LPS/AlCl_3_ injection as markers of inflammation, cholinergic activity, and oxidative stress. Furthermore, the interaction of two main components, 11,14,17-eicosatrienoic acid and phytol, was investigated by molecular docking, with the important anti-inflammatory targets nuclear factor kappa B (NF-κB) and cyclooxygenase 2 (COX-2). Specifically, the absorption, distribution, metabolism, excretion, and toxicity (ADMET) and drug-likeness properties of HFFO were studied by ADMETlab.

**Results:**

The results showed that HFFO reduced cognitive deficits in zebrafish T-maze induced by LPS/AlCl_3_. While the LPS/AlCl_3_ treatment increased MDA content, lowered ACh levels in the zebrafish brain, and elevated levels of central and peripheral proinflammatory cytokines, these effects were reversed by 100 mg/kg HFFO except for MDA. Moreover, 11,14,17-eicosatrienoic acid and phytol showed a good affinity with NF-κB, COX-2, and HFFO exhibited acceptable drug-likeness and ADMET profiles in general.

**Conclusion:**

Collectively, this study's findings suggest HFFO as a potent neuroprotectant, potentially valuable for the prevention of memory impairment caused by cholinergic deficiency and neuroinflammation.

## Introduction

Neurodegenerative disorders (NDs) are chronic, progressive, and severely debilitating neurological illnesses caused by the loss of neurons in the brain and spinal cord (Whitehouse et al., 1982). With the aging of the population and the deterioration of the environment, the threat of NDs to human beings is becoming increasingly serious, among which the most representative is Alzheimer's disease (AD). Existing studies have shown that NDs are closely related to neurodegeneration and neuronal damage caused by neuroinflammation in the brain (Uttara et al., 2009; Kumar et al., 2016; Ransohoff, 2016). However, current clinical drugs for the prevention and treatment of NDs have limited efficacy, such as donepezil, which can only partially relieve symptoms but not reverse or prevent their development (Bachurin et al., 2017). Thus, daily nutritional interventions such as healthy lipid intake are increasingly viewed as important supplementary approaches to drugs for these chronic neurodegenerative diseases in a specific population (Petersson and Philippou, 2016; McGrattan et al., 2019; Livingston et al., 2020).

Marine organisms live in a water environment with low temperatures, featuring them with a rich level of polyunsaturated fatty acids (PUFAs) synthesized by themselves or accumulated from their food chains. Many PUFAs possess anti-neuroinflammatory effects and show potential for preventing or treating NDs. For example, docosahexaenoic acid (DHA) or its metabolites can inhibit acetylcholinesterase (AChE), promote neuronal survival/growth and synaptogenesis, increase cortical brain-derived neurotrophic factor (BDNTF), inhibit oxidative-stress-induced caspase-3 activation and IL-1-stimulated expression of COX-2, and protect liposaccharide (LPS)-induced mouse model of acute neuroinflammation (Barbosa et al., 2014). Eicosapentaenoic acid (EPA) ethyl ester was reported to lower interleukin-1β (IL-1β) and increase peroxisome proliferator-activated receptor (PPAR)γ in Aβ i.c.v. aged mice and EPA was also found to be the active principle of a microalgae *Nannochloropsis oceanica*, demonstrating anti-inflammatory, antioxidative, and anti-amyloidogenesis activities in a mouse model of LPS-induced AD (Trépanier et al., 2016; Choi et al., 2017). Currently, the main commercial sources for marine PUFAs are fish and microalgae; however, macroalgae should not be neglected considering their high proportion of PUFAs in their total lipids and neuroprotective isoprenoids like fucoxanthin coexisting in lipids (Barbalace et al., 2019; Catanesi et al., 2021).

Brown alga *Hizikia fusiforme* (also named “*Hijiki*” or *Sargassum fusiforme*), belonging to the class Phaeophyceae, order Fucales, and family Sargassaceae (Meinita et al., 2021), is a kind of natural healthy food that can be used as both medicine and food. Its medicinal effects are clearly recorded in traditional Chinese medicinal works, including Shennong Materia Medica Classic (dated 200 AD) and Compendium of Materia Medica (published in the late 16th century). As a delicious food, it is now popular in China, Korea, Japan, the United Kingdom, and North America and has been effectively cultivated in China and Korea (Liu et al., 2020; Meinita et al., 2021). In recent years, it has attracted more attention from natural medicine research (Liu et al., 2020; Meinita et al., 2021). Researchers have found a lot of active ingredients in it that have the potential to improve learning and memory (Hu et al., 2016; Bogie et al., 2019). *Sargassum fusiforme* polysaccharides (SFPS) can inhibit the apoptosis of central nerve cells, increase the activities of total antioxidant capacity (T-AOC) and superoxide dismutase (SOD) in serum, and reduce the content of malonaldehyde (MDA), thus improving the serum antioxidant ability of mice and enhancing the learning and memory ability of mice (Liu et al., 2018). In addition, SFPS significantly inhibited the production of TNF-α and other inflammatory molecules, and it can significantly reduce reactive oxygen species (ROS), cell death, and NO levels in a dose-dependent manner in LPS-induced zebrafish (Wang et al., 2021). *Sargassum fusiforme* polyphenols (SFP) can reduce brain damage and delay the occurrence of AD mainly through their antioxidant effect (Hu et al., 2016). In addition, the *Hizikia fusiforme* fucoxanthin showed strong scavenging ability on 1, 1-diphenyl-2- picrylhydrazyl (DPPH), OH^−^, O_2_, and H_2_O_2_ (Kuang and Zhang, 2014). But the study is rare on the brain-health value of its oil. Previously, we reported that the *Hizikia fusiforme* functional oil (HFFO) contained arachidonic acid (ARA, one of the two main PUFAs in the brain) (20.54%), 11,14,17-eicosatrienoic acid (ETrA) (19.53%), phytol (6.55%) (Bazinet and Layé, 2014), and other molecules displayed *in vitro* acetylcholinesterase inhibitory and nitroxide scavenging activity (Yang et al., 2020). However, the *in vivo* effect remains unclear.

Bacterial LPS or AlCl_3_ can cause AD-like pathology, aggravating neuroinflammation, oxidative stress, and acetylcholinesterase (AChE) activity in the brain of rodents (Tyagi et al., 2010; Mathiyazahan et al., 2015; Ahmad Rather et al., 2019; Huat et al., 2019). They also affect adult zebrafish *via* similar mechanisms or cause-related responses (Senger et al., 2011; Gonçalves et al., 2012). Here, we develop LPS/AlCl_3_-induced neuroinflammatory zebrafish model to assess the potential effects of HFFO on memory and cognitive impairment *in vivo* and its drug-related properties *in silico*. The putative neuroprotective activity of HFFO in zebrafish was also probed by evaluating MDA and ACh levels in the brains and by analyzing the levels of the IL-1β and TNF-α in both brain and peripheral tissues.

## Materials and methods

### Animals

Wild-type AB zebrafish of 6–8 months old (adult, 1:1 male to female) were purchased from Shanghai Jianyu Aquarium (Shanghai, China) and acclimatized for at least 2 weeks in a 50-L tank in the aquatic facility of Guangdong Ocean University Shenzhen Research Institute. The fish were raised according to standard conditions (the illumination condition is a 14-h light/10-h dark cycle and the ambient temperature is 25 ± 2°C) (Westerfield, 2007) and fed with *Artemis* larvae at 9 am and 2 pm every day.

### Chemicals

*Hizikia fusiforme* essential oil (HFFO) was prepared by our research group (Yang et al., 2020). The main ingredients of HFFO are arachidonic acid, 11,14,17-eicosatrienoic acid, palmitic acid, and phytol. The BCA protein, Ach and MDA assay kits, fish IL-1β enzyme-linked immunosorbent assay (ELISA) kit, and fish TNF-α ELISA kit were purchased from Nanjing Jiancheng Bioengineering Institute (Nanjing, China). Eugenol was purchased from Huaxia Reagent (Chengdu, China) and LPS from Shanghai Ryon Biological Technology Co. Ltd. (Shanghai, China). All the chemicals involved in this study are analytical grade.

### Animal model development and T-maze behavioral testing

To perform the experiments based on the LPS model, 50 zebrafish (3.0 ± 0.4 cm in length) were randomly divided into a control group, an LPS model, and three treatment groups (n = 10 per group). The treatment groups received 50, 100, or 200 mg/kg HFFO with food containing HFFO for 2 weeks. The control and LPS groups were fed equal amounts of normal food. After 2 weeks, the LPS and HFFO groups were anesthetized and injected intraperitoneally (i.p.) with LPS solution (0.015 mg/ml, 5 μl) using a 10-μl gas phase injection needle 0.5 mm in the outer diameter. The control group was injected with the same amount of saline. Memory testing was performed in the T-maze 24 h later. Before the injection of LPS/saline, the fish were previously trained in the T-maze for 4 days to record their latency of entering the enriched chamber (EC) zones for the first time.

The experiments on the AlCl_3_ model were developed similar to the LPS model and involved a 20-day feeding with normal or HFFO-containing food, followed by an i.p. injection of the AlCl_3_ solution (4.2 mg/ml, 5 μl, pH = 5.0 ± 0.2). The control group was injected with the same amount of saline and tested in the T-maze 24 h later.

In this study, we performed a zebrafish cognitive test using an aquatic T-maze (Tyagi et al., 2010). Apowersoft (Apowersoft Co. Ltd., Hong Kong, China) was used to record videos from Microsoft LifeCam Studio 1080p HD cameras. Offline video analyses were performed by the Supersys software (Xinruan, Shanghai, China), assessing the latency of zebrafish entering the enriched chamber (EC) zone for the first time.

### Molecular biomarker assays

After 24 h of behavioral testing, the fish were euthanized to prepare the brain and peripheral tissue homogenates (Nie et al., 2022). Zebrafish brain sample supernatants were used to estimate tissue biomarker changes, i.e., Ach and MDA levels. In addition, Il-1 β and TNF-α levels in the supernatant of the brain and peripheral tissues of zebrafish were determined according to the ELISA kits' instructions (Rishitha and Muthuraman, 2018).

### Statistical analysis

Statistical analysis was performed by one-way analysis of variance (ANOVA, factor: group) followed by a *post hoc* Dunnett for significant AVOVA data. The results are expressed as the mean ± SD. *P*-value was set at <0.05 for all tests.

### Molecular docking analysis

This study employed AutoDock Vina (Trott and Olson, 2010) for the *in silico* protein–ligand docking analysis. Protein three-dimensional (3D) structures (PDB format) of NF-κB (PDB: 1VKX) and COX-2 (PDB: 5IKT) were retrieved from Protein Databank (PDB) (http://www.rcsb.org). Chemical 3D conformer of the compounds (phytol and eicosatrienoic acid) was obtained from the PubChem database (http://www.ncbi.nlm.nih.gov/pccompound). Molecular docking simulations were performed using AutoDock Vina using default parameters. The protein–ligand interactions were investigated in the PyMOL (https://www.PyMOL.org).

### *In silico* prediction of ADMET and drug-likeness properties of HFFO

We used ADMETlab 2.0 (https://admetmesh.scbdd.com/pub/) to assess HFFO pharmacokinetic characteristics and toxicity; the online platform can help researchers predict the absorption, distribution, metabolism, excretion, and toxicity (ADMET) and drug-likeness properties of compounds (Guo et al., 2021).

## Results

### HFFO improved LPS/AlCl_3_-induced memory impairment

**Figure 1:**
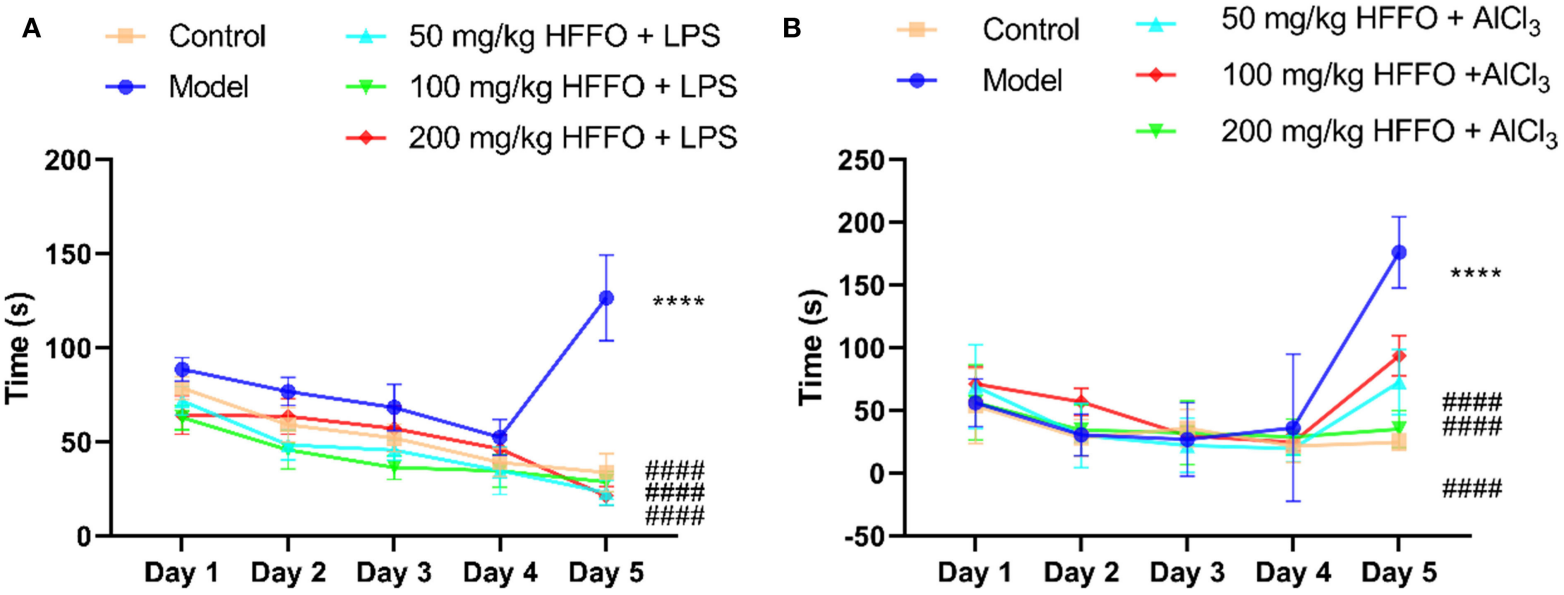
The latency (s) of first entry into the enriched chamber (EC) zone of the T-maze test. LPS model **(A)**, AlCl3 model **(B)**. *n* = 10, ^*^p < 0.05, ^**^p < 0.01, ^***^p < 0.005, ^****^p < 0.001, vs. the control group; #*p* < 0.05, ##*p* < 0.01, ###*p* < 0.005, ####*p* < 0.001, vs. the model group.

Overall, zebrafish treated with LPS/AlCl_3_ showed impaired spatial memory in the T-maze test; there were significant treatment effects for zebrafish cognitive performance by HFFO treatment (*F*(4, 45) = 141.9, *P* < 0.001 in Figure 1A; F(4, 45) = 80.63, *P* < 0.001 in Figure 1B).

**Figure 2:**
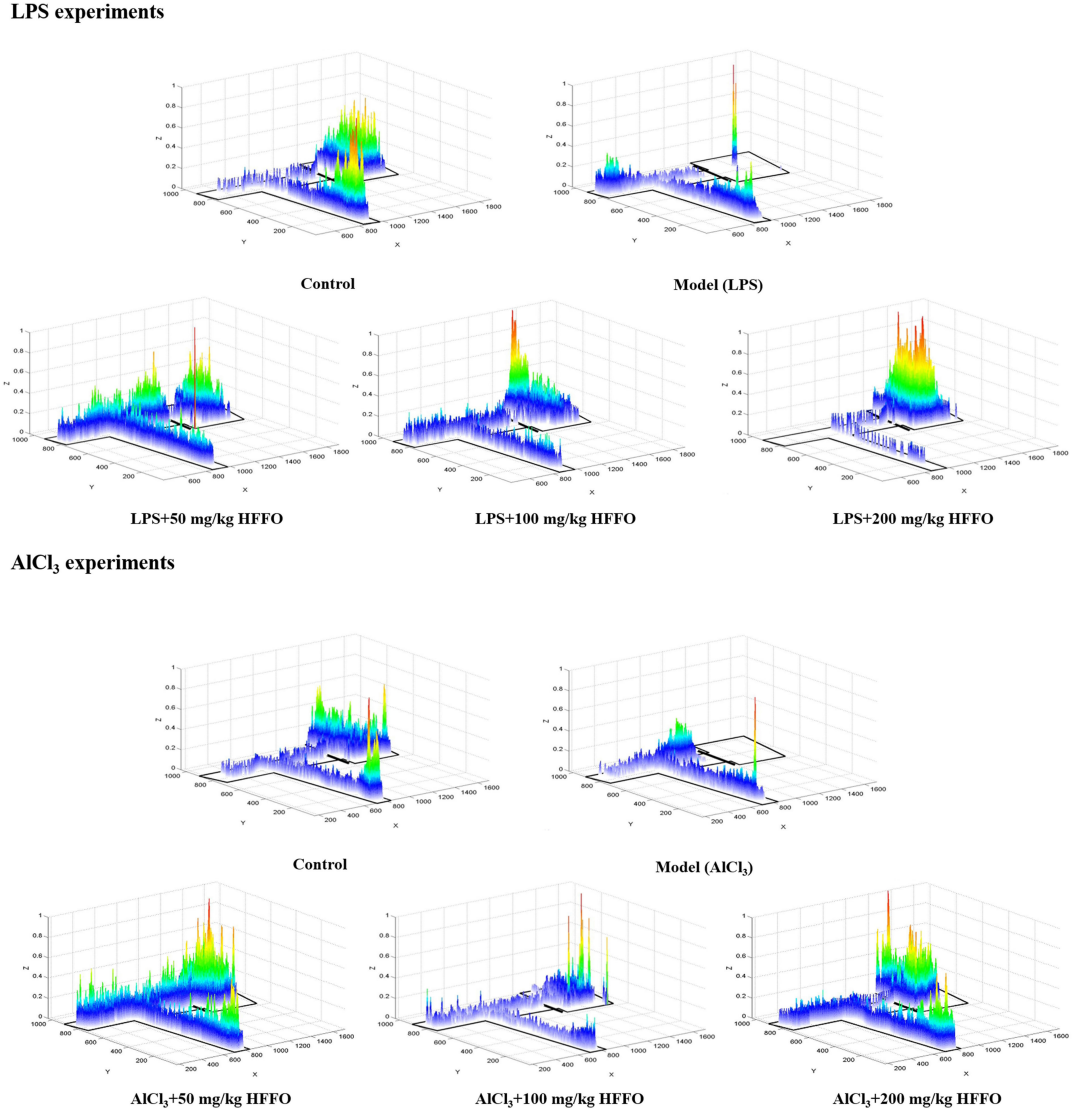
Heatmaps of zebrafish activity in the T-maze on the fifth day. The X-axis and Y-axis in the figure represent the motion trajectory of zebrafish, while the Z-axis represents the residence time of zebrafish. The higher the Z-axis, the longer the residence time of zebrafish at a certain point.

Subsequent *post hoc* testing revealed that the latency of first entry into the EC zone increased for the model groups. The swimming tracks also clearly showed the reduced preference of the model group fish to the EC zone (Figure 2) following the LPS/AlCl_3_ injections. In contrast, pretreatment with low to high doses of HFFO prevented these effects of LPS/AlCl_3_.

### Effect of HFFO against LPS/AlCl_3_ on proinflammatory cytokines

**Figure 3:**
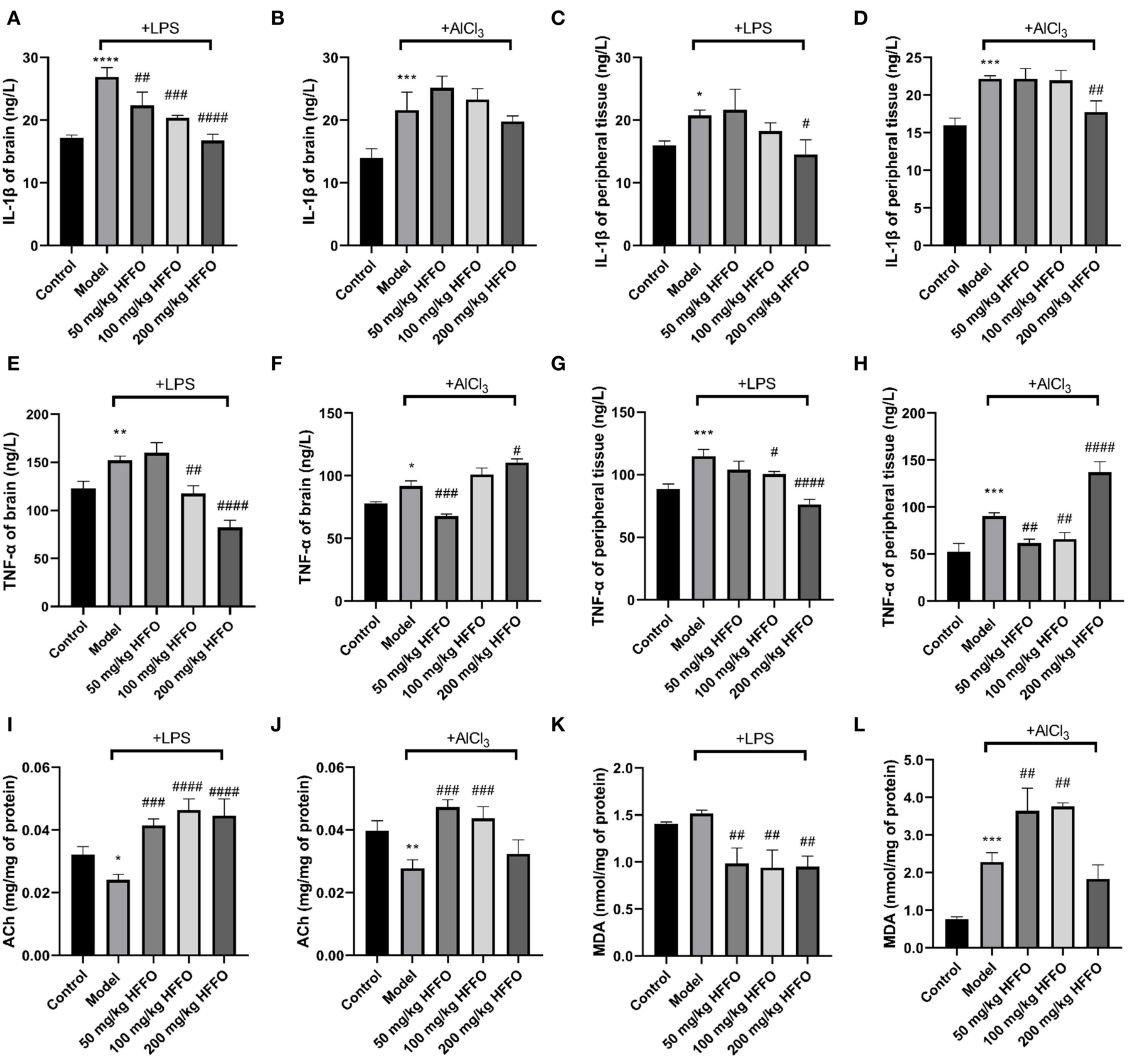
IL-1β **(A–D)** and TNF-α **(E–H)** content in zebrafish brain and peripheral tissue, and ACh **(I,J)** and MDA **(K,L)** levels in zebrafish brain tissue. n = 3, ^*^p < 0.05, ^**^p < 0.01, ^***^p < 0.005, ^****^p < 0.001, vs. the control group; #*p* < 0.05, ##*p* < 0.01, ###*p* < 0.005, ####*p* < 0.001, vs. the model group.

In addition to overt behavioral effects, the treatment with LPS/AlCl_3_ promoted the release of inflammatory cytokine IL-1β from the brain and peripheral tissues (Figures 3A–D), and HFFO supplementation reduced LPS-induced IL-1β significantly at all and part of the doses in the brain and peripheral tissues, respectively (*F*(4, 10) = 32.93, *P* < 0.001 in Figure 3A; *F*(4, 10) = 7.160, *P* < 0.01 in Figure 3C). As for AlCl_3_-caused IL-1β increase, HFFO showed a reduction in peripheral tissue at the high dose while no inhibition in the brain at the tested doses (F(4, 10) = 15.69, *P* < 0.001 in Figure 3B; F(4, 10) = 19.28, *P* < 0.001 in Figure 3D).

Besides, the treatment with LPS/AlCl_3_ also promoted the release of TNF-α from brain and peripheral tissues, and HFFO supplementation inhibited these effects at part of the doses (F(4, 10) = 46.81, *P* < 0.001 in Figure 3E; F(4, 10) = 84.67, *P* < 0.001 in Figure 3F; F(4, 10) = 30.44, *P* < 0.001 in Figure 3G; F(4, 10) = 62.92, *P* < 0.001 in Figure 3H).

### Effect of HFFO against LPS/AlCl_3_ on ACh and MDA

Furthermore, paralleling their cognitive deficits in the T-maze, zebrafish treated with LPS/AlCl_3_ decreased brain ACh content (i.e., exhibited higher brain AChE activity), whereas almost all the doses of HFFO significantly reversed this phenomenon (F (4, 10) = 23.93, *P* < 0.001 in Figure 3I; F (4, 10) = 17.16, *P* < 0.001 in Figure 3J). This is consistent with the fact that excessive AChE activity is closely related to memory deficits (Giacobini et al., 2002).

Because oxidative stress plays a key role in neuronal injury and apoptosis (Mcbean et al., 2016), the levels of MDA (a lipid peroxidation degradation indicator) were also analyzed to assess the oxidant–antioxidant balance in the zebrafish brain, to parallel their behavioral analyses. In the LPS model, HFFO significantly reduced MDA levels in the brain of zebrafish. However, in the AlCl_3_ model, low and medium doses of HFFO significantly increased MDA levels (F (4, 10) = 15.40, *P* < 0.001 in Figure 3K; F (4, 10) = 42.12, *P* < 0.001 in Figure 3L).

### Molecular docking and interaction analysis

**Figure 4:**
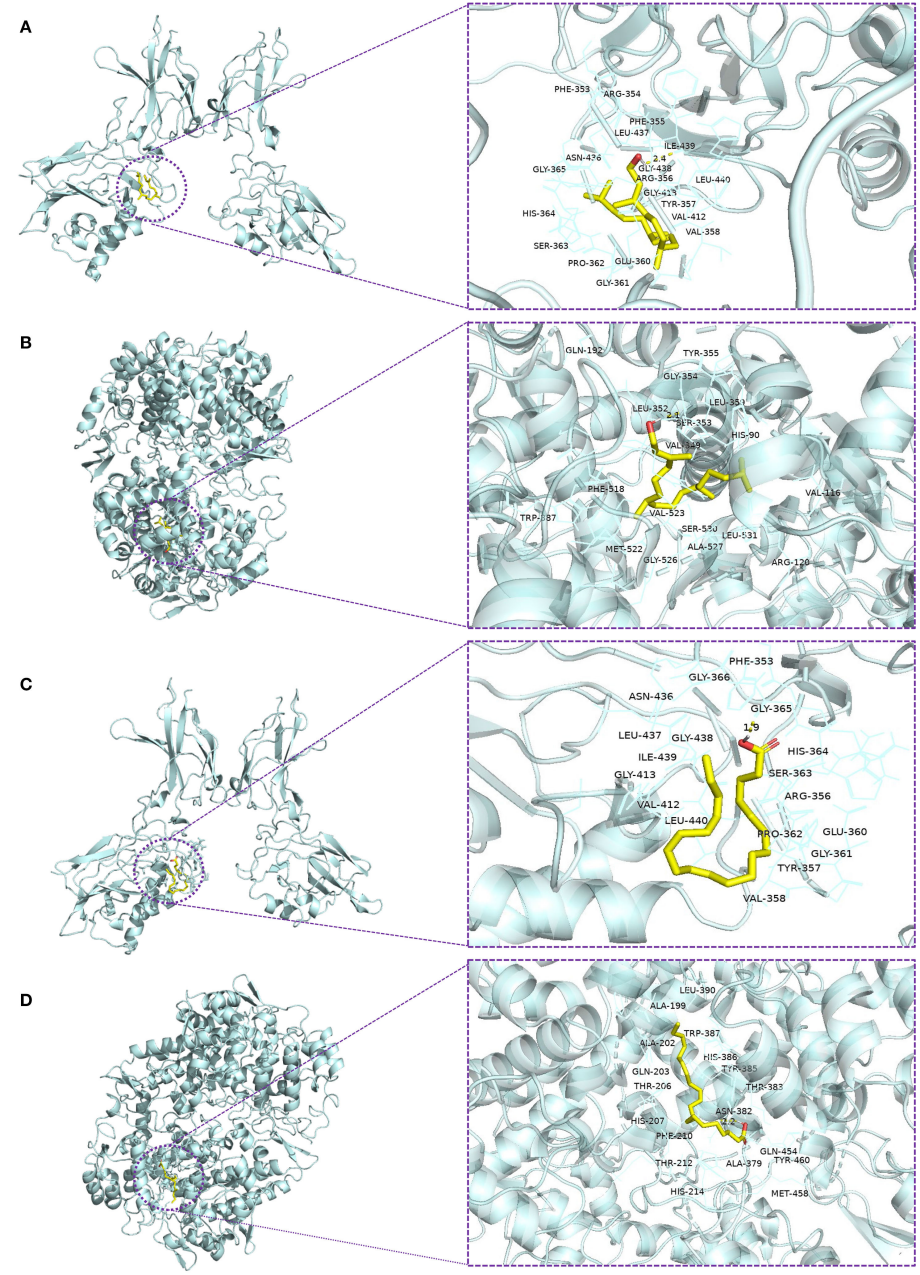
Molecular docking of ligands with their potential targets. Docking interaction 3D view of phytol with NF-κB **(A)** and COX-2 **(B)**, and eicosatrienoic acid with NF-κB **(C)** and COX-2 **(D)**. Residues are within the distance of 4Å, and the yellow dotted lines indicate hydrogen bonds.

Previous studies have suggested that phytol and eicosatrienoic acid may exert anti-inflammatory effects by interacting with nuclear factor kappa B (NF-κB) (Tak and Firestein, 2001; Silva et al., 2014; Chen et al., 2015) and cyclooxygenase 2 (COX-2) (Islam et al., 2020; Baker et al., 2021). In our study, eicosatrienoic acid and phytol are two major components of the HFFO. To explore the potential interactions and gain insights into the mechanism of phytol with NF-κB and COX-2, and eicosatrienoic acid with NF-κB and COX-2, molecular docking studies were conducted. Phytol displayed considerable binding affinity to NF-κB and COX-2 at −6.1 and −7.2 kcal/mol (Table 1), respectively. The interaction forces between phytol and potential targets (NF-κB and COX-2) mainly include polar (hydrogen bonds) interactions and non-polar interactions (Figures 4A,B). Specifically, phytol forms a hydrogen bond with NF-κB at Ile439 (length of 2.4 Å) and non-polar interactions with NF-κB at Tyr357, Val412, Val358, etc. (Figure 4A). Phytol forms a hydrogen bond with COX-2 at Ser353 (length of 2.1 Å) and non-polar interactions with COX-2 at Tyr355, Gly354, His90, etc. (Figure 4B). In terms of eicosatrienoic acid, it also showed considerable binding affinity to NF-κB and COX-2 at −6.1 and −6.8 kcal/mol (Table 1), respectively. The interaction forces were similar to those of the phytol with NF-κB and COX-2 (Figures 4C,flreffig4D). In summary, these results show that phytol and eicosatrienoic acid bind to the analyzed targets with high affinity and form clearly defined specific interactions with them.

**Table 1:** Molecular docking affinity (kcal/mol) of phytol and eicosatrienoic acid with NF-κB and COX-2, respectively.

**Ligand**	**Protein**	**PDB entry**	**Affinity (kcal/mol)**
Phytol	NF-κB	1VKX	−6.1
Phytol	COX-2	5IKT	−7.2
Eicosatrienoic acid	NF-κB	1VKX	−6.1
Eicosatrienoic acid	COX-2	5IKT	−6.8

### Prediction of ADMET and drug-likeness properties

To investigate the pharmacokinetic profile of HFFO and its potential as a drug, we used ADMETlab 2.0 (Jiang et al., 2020; Guo et al., 2021) to predict its ADMET (absorption, distribution, metabolism, elimination, and toxicity) and drug-likeness properties. The corresponding results are presented in Table 2. Generally, HFFO possesses acceptable ADMET and drug-likeness properties as oral drugs. Its main components, including arachidonic acid, eicosatrienoic acid, palmitic acid, tetradecanoic acid, and 9-hexadecenoic, were endowed with promising activities in MDCK permeability, human intestinal absorption (HIA), and blood–brain barrier (BBB) penetration. The compounds also exhibited acceptable safety profiles, generally performing well on most metrics (e.g., low hERG blockers (index of heart toxicity), human hepatotoxicity (H-HT), and rat oral acute toxicity), and these components fulfilled one or more of the drug-likeness criteria, such as the Lipinski rule (Lipinski et al., 2001), Pfizer rule (Hughes et al., 2008), and golden triangle (Johnson et al., 2009). In addition, the physical properties of the main HFFO ingredients are within an acceptable range (Supplementary Tables S1–S6). In summary, the predicted ADMET properties of the main components within HFFO indicate that they are promising candidates as lead compounds.

**Table 2:** ADMET and drug-likeness properties of HFFO main ingredient through online prediction tool of ADMETlab 2.0.

	**Arachidonic acid**		**Eicosatrienoic acid**		**Palmitic acid**		**Tetradecanoic acid**		**Phytol**		**9-Hexadecenoic acid**	
**Property**	**Value**	**Decision**	**Value**	**Decision**	**Value**	**Decision**	**Value**	**Decision**	**Value**	**Decision**	**Value**	**Decision**
**Absorption**												
Caco-2 permeability (log cm/s)	−5.263	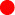	−5.168	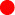	−5.027	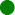	−4.960	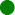	−4.513	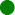	−5.059	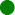
MDCK permeability (cm/s)	7.8e−05	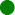	9.3e−05	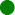	2.5e−05	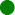	2.8e−05	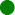	1.3e−05	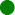	3.3e−05	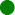
Pgp–inhibitor	0.000	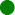	0.000	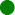	0.009	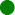	0.0280	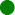	0.049	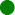	0.009	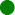
Pgp–substrate	0.000	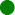	0.000	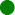	0.000	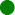	0.000	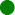	0.002	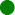	0.000	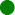
HIA	0.033	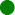	0.027	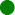	0.005	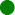	0.005	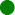	0.003	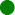	0.015	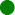
F_20%_	1.000	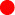	1.000	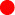	0.644	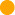	0.754	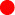	0.848	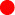	0.828	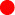
**Distribution**												
PPB	0.999	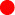	0.993	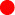	0.990	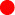	0.982	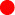	0.989	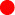	0.997	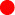
VD (L/kg)	0.735	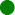	0.912	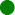	0.608	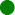	0.416	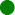	2.298	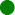	0.503	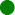
BBB penetration	0.002	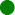	0.005	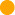	0.060	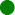	0.163	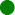	0.2	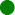	0.0310	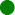
Fu	0.001	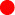	0.000	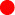	0.001	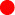	0.001	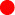	0.002	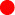	0.0006	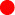
**Metabolism**												
CYP1A2–inhibitor	0.205	–	0.241	–	0.300	–	0.256	–	0.387	–	0.251	–
CYP1A2–substrate	0.857	+	0.638	+	0.194	–	0.202	–	0.192	–	0.196	–
CYP2C19–inhibitor	0.114	–	0.168	–	0.203	–	0.099	–	0.437	–	0.101	–
CYP2C19–substrate	0.166	–	0.118	–	0.110	–	0.213	–	0.072	–	0.090	–
CYP2C9–inhibitor	0.272	–	0.299	–	0.174	–	0.249	–	0.383	–	0.296	–
CYP2C9–substrate	0.991	+	0.991	+	0.989	+	0.986	+	0.863	+	0.991	+
CYP2D6–inhibitor	0.149	–	0.110	–	0.008	–	0.007	–	0.197	–	0.018	–
CYP2D6–substrate	0.939	+	0.921	+	0.054	–	0.069	–	0.037	–	0.129	–
CYP3A4–inhibitor	0.100	–	0.130	–	0.024	–	0.017	–	0.262	–	0.036	–
CYP3A4–substrate	0.070	–	0.048	–	0.019	–	0.024	–	0.116	–	0.022	–
**Excretion**												
CL (mL/min/kg)	3.007	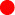	3.084	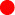	2.377	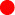	2.303	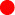	5.653	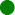	2.373	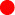
T_1/2_	0.916	–	0.887	–	0.610	–	0.718	–	0.210	–	0.827	–
**Toxicity**												
hERG blockers	0.026	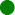	0.027	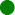	0.056	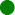	0.040	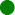	0.010	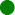	0.036	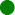
H–HT	0.251	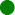	0.281	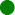	0.026	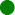	0.030	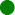	0.046	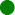	0.039	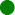
DILI	0.006	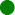	0.014	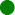	0.043	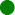	0.042	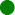	0.064	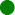	0.018	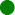
Ames toxicity	0.945	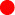	0.604	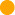	0.005	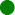	0.006	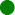	0.001	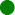	0.002	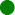
Rat oral acute toxicity	0.003	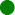	0.012	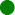	0.029	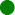	0.037	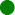	0.012	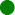	0.018	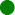
FDAMDD	0.349	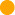	0.206	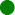	0.015	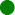	0.015	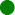	0.666	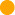	0.023	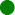
Skin sensitization	0.959	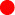	0.953	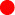	0.899	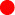	0.807	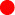	0.970	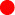	0.912	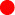
Carcinogenicity	0.783	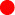	0.557	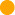	0.064	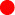	0.078	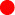	0.021	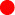	0.050	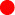
Eye corrosion	0.147	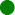	0.414	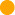	0.977	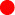	0.979	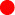	0.851	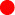	0.966	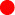
Respiratory toxicity	0.855	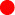	0.845	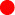	0.891	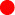	0.848	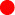	0.042	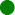	0.805	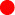
**Drug-likeness**												
MCE-18 (Ivanenkov et al., 2019)	0.000	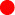	0.000	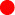	0.000	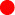	0.000	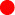	4.000	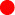	0.000	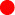
Lipinski rule (Lipinski et al., 2001)	accepted	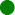	accepted	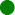	accepted	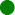	accepted	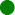	accepted	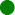	accepted	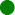
Pfizer rule (Hughes et al., 2008)	accepted	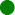	rejected	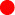	rejected	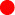	rejected	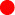	rejected	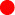	rejected	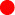
Golden triangle (Johnson et al., 2009)	accepted	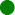	accepted	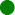	accepted	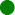	rejected	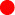	rejected	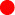	rejected	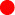
GSK rule (Guo et al., 2021)	accepted	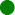	rejected	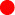	rejected	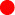	accepted	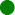	rejected	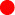	accepted	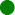

•: Excellent, •: medium, •: bad.

## Discussion

Mounting evidence shows that peripheral administration of LPS in mice can activate astrocytes and microglia; increase the expression of COX-2, inducible nitric oxide synthase (i-NOS), and proinflammatory cytokines; promote the intracellular accumulation of amyloid precursor protein, amyloid β (Aβ) protein, and hyperphosphorylated tau; and finally exacerbate memory deficits (Catorce and Gevorkian, 2016). In addition, LPS potently induces oxidative stress in the rodent brain and impairs memory cognition (Cunningham and Sanderson, 2008; Tyagi et al., 2008; Czerniawski and Guzowski, 2014; Ming et al., 2015). In addition, LPS administration has also been linked to enhanced brain AChE activity following an intracerebroventricular or systemic administration of LPS in rodents (Sebai et al., 2009; Han et al., 2019).

Likewise, subchronic exposure of zebrafish to AlCl_3_ or feeding of AlCl_3_ in mice enhances brain AChE activity (Zatta et al., 2002; Senger et al., 2011; Liu et al., 2013). Intriguingly, activated AChE can deteriorate Aβ aggregation, decrease brain-derived neurotrophic factor (BDNF) expression (Auti and Kulkarni, 2019), and further promote oxidative stress and neuroinflammation through a ‘cholinergic anti-inflammatory pathway’ (CAIP) in which α7 nicotinic acetylcholine (ACh) receptor acts as an effector player (Tabet, 2006; Benfante et al., 2021). Another study also reported that 10–20 days of chronic exposure of adult zebrafish to aluminum leads to brain oxidative stress and behavioral disorders (Capriello et al., 2021).

In this study, i.p. injection of LPS/AlCl_3_ in zebrafish has successfully induced acute inflammatory responses peripherally and centrally and enhanced brain AChE activity and oxidative stress. Importantly, acute i.p. administration of LPS/AlCl_3_ strongly impaired the spatial and contextual memory of zebrafish in the T-maze test. Collectively, these findings are generally consistent with previous evidence that LPS and other proinflammatory factors, as well as AlCl_3_, induce memory deficits in vertebrates (White et al., 1992; Hauss-Wegrzyniak et al., 1998, 2000; Rosi et al., 2003, 2004, 2005; Senger et al., 2011; Lee et al., 2018; Huang et al., 2019). Thus, two zebrafish memory deficit models have been established, induced by LPS and AlCl_3_, respectively. Their memory impairment mechanisms involve cholinergic, inflammatory, and oxidative-stress systems.

In these two models, HFFO supplementation reversed LPS/AlCl_3_-induced memory deficits, exhibiting its suppression of AChE activity and oxidative stress in the brain as well as central and peripheral inflammation. Supplementation with HFFO potently inhibited acute central and peripheral inflammation in the LPS-treated zebrafish. We speculate that this may be partially related to the underlying mechanism of the anti-inflammatory effect of phytol, which interacts with nuclear factor kappa B (NF-κB) to reduce interleukin-1beta (IL-1 β) and tumor necrosis factor (TNF-α) levels (Tak and Firestein, 2001; Silva et al., 2014). In addition, phytol can inhibit cyclooxygenase 2 (COX-2) *via* suppressing IL-1β and NF-κB (Islam et al., 2020). Besides, eicosatrienoic acid may also contribute to the anti-inflammatory effect of HFFO because some studies suggest eicosatrienoic acid has an anti-inflammatory role by suppressing NF-κB (Chen et al., 2015), and eicosatrienoic acid (all cis-7,-11,-14 20:3) can inhibit COX-2 (Baker et al., 2021). Moreover, the docking results of phytol with NF-κB and COX-2 and eicosatrienoic acid with NF-κB and COX-2 in this study also support this hypothesis to some extent. COX-2 is an important enzyme that catalyzes the conversion of arachidonic acid to prostaglandins. Under the condition of inhibition of COX-2 activity, arachidonic acid will be accumulated or converted to epoxyeicosatrienoic acid (EETs) by lipoxygenase and cytochrome P450 (CYP) epoxygenases (Strauss, 2008). EETs play a protective role in peripheral tissues by modulating a variety of cellular signaling pathways, and these effects may contribute to neuroprotection (Lakkappa et al., 2016). Treatment of AlCl_3_ and 200 mg/kg HFFO showed a significant pro-inflammatory effect compared with the treatment of AlCl_3_. We speculate that this may be because AlCl_3_ causes severe oxidative damage to neurons, and HFFO does not show a significant ability to alleviate such oxidative damage, which may further aggravate the inflammatory response. HFFO has a poor effect on such severe oxidative damage-inflammation in general. In addition, an excessive supply of exogenous arachidonic acid affected the inhibitory effect of phytol and eicosatrienoic acid on COX-2. This leads to the increased levels of prostaglandins produced by COX-2, which are linked to inflammation (Hamilton et al., 1999). However, HFFO has a better effect on LPS-induced inflammation.

The inhibition of brain AChE elevates ACh levels and hence may positively affect cognitive function (Scarpini et al., 2016). In this study, supplementation with low and medium doses of HFFO also dramatically reversed the ACh decrease in the LPS/AlCl_3_-treated zebrafish (Figure 3). This is consistent with our previous *in vitro* study that HFFO possesses *in vitro* AChE inhibitory activity and it mainly comes from ARA and ETrA (Yang et al., 2020). Another study also supports the AChE inhibitory activity of ARA (Ahmed et al., 2011).

Besides, MDA levels in lipid peroxidation degradation products reflect the level of lipid peroxidation *in vivo* and indirectly reflect the cellular damage and oxidative stress in brain tissue (Leutner et al., 2001; Long et al., 2009). In this study, MDA brain levels showed no significant change, 24 h after the LPS administration, which was speculated to be caused by a too low dose of LPS. However, MDA content in the low-, medium-, and high-dose HFFO groups decreased significantly, indicating that HFFO can reduce LPS-caused oxidative stress by inhibiting lipid peroxidation mildly. The antioxidant activity of HFFO may be related to the antioxidant mechanism of ARA or its metabolite EETs and phytol (Liu et al., 2011; Silva et al., 2014; Akimov et al., 2020). After 24 h of the injection of AlCl_3_, MDA levels increased. This is consistent with the increase in lipid peroxidation reported in the literature (Capriello et al., 2021). However, supplementation with HFFO in low and medium doses significantly increased MDA levels, while a high dose of HFFO showed no significantly lower MDA level compared to the model group. It is supposed that HFFO only possesses mild antioxidant activity, as it displayed in our previous *in vitro* study on LPS-induced BV-2 microglial cell lines (Yang et al., 2020) and this study on LPS-induced zebrafish. So, low and medium doses of HFFO can inhibit low-level lipoperoxidation caused by LPS but are not sufficient to counter high-level lipoperoxidation caused by aluminum. However, the abnormal MDA increase compared to the model group may be due to the unclear interaction between AlCl_3_ and HFFO, which remains to be further investigated in the future.

The assessment of pharmacological properties of compounds is deemed to be a crucial step in drug discovery (Cumming et al., 2013; Neggers et al., 2018). On account of the time and expense required to perform *in vivo* estimations, *in silico* approaches have become indispensable elements for drug discovery (Daina et al., 2017; Göller et al., 2020; Guo et al., 2021). Our study employed ADMETlab2.0, a multitask graph attention framework-based method, for the prediction of absorption, distribution, metabolism, excretion, and toxicity of the compounds (Guo et al., 2021). *In silico* prediction indicated that the main components of HFFO possessed acceptable ADMET properties, were capable of crossing the BBB, and obeyed one or more of the drug-likeness criteria. Among them, the arachidonic acid (ARA), the highest proportion of HFFO, showed the best performance in drug-likeness by respecting the Lipinski rule, Pfizer rule, golden triangle, and GSK rule. In addition, ARA, as well as docosahexaenoic acid (DHA), is a major polyunsaturated fatty acid in the human brain (Sun et al., 2018). The uptake of ARA in the human brain was generally greater than the uptake of DHA. After esterification *in vivo*, ARA is stored in the phospholipids of the cellular membranes and plays an important role in signal transduction by modulating membrane dynamics and activating receptors. Moreover, ARA is an important precursor of anandamide (AEA) and 2-arachidonoylglycerol (2-AG), which are regulators of synaptic neurotransmitter release (Busquets-Garcia et al., 2011). The aforementioned features render ARA a promising candidate for applying in the field of NDs. Notably, the main components of HFFO were predicted to carry a risk of skin sensitization and respiratory toxicity, which needs to be validated in further work. In addition, ARA was predicted to be a substrate of CYP1A2, CYP2C9, and CYP2D6. Therefore, it may be essential to avoid co-administration with CYP1A2, CYP2C9, and CYP2D6 inhibitors.

Recently, many studies have found that *Hizikia fusiforme* extracts have the function of improving learning and memory (Kuang and Zhang, 2014; Liu et al., 2018; Wang et al., 2021). Here, we found that its oil HFFO prevents cognitive deficits (induced by LPS/AlCl_3_ in zebrafish models) and exerts neuroprotective effects by anti-neuroinflammation and inhibition of acetylcholinesterase activity.

## Conclusion

Collectively, the findings suggest HFFO as a potent neuroprotectant potentially valuable for the prevention of memory impairment caused by cholinergic deficiency and neuroinflammation.

## Data availability statement

The original contributions presented in the study are included in the article/Supplementary material, further inquiries can be directed to the corresponding author.

## Ethics statement

The animal study was reviewed and approved by the Animal Ethics Committee of Guangdong Ocean University, numbered 2020-6-20-1.

## Author contributions

Y-YN and L-JZ performed the zebrafish experiment, calculation study, analyzed the data, and wrote the original draft. W-CY, Y-ML, X-XM, Z-YY, and Y-PZ prepared the compound sample and assisted in zebrafish experiments. YZ designed, guided the experiments, provided critical comments for the research, and revised the study. P-ZH and Y-YL provided critical comments during the research and polished the study. All authors have read and approved the final manuscript.

## Funding

This study was funded by the special project in key fields of Guangdong Provincial Higher Education Institutions (Biomedicine and Health Care) with grant number (2021ZDZX2064), the Natural Science Foundation of Guangdong Province (2022A1515010783), the Basic Research Project of Shenzhen Science and Technology Innovation Commission with grant number (JCYJ20190813105005619), the Shenzhen Dapeng New District Scientific and Technological Research and Development Fund with grant number (KJYF202001-07), and the Innovation and Development Project about Marine Economy Demonstration of Zhanjiang City (XM-202008-01B1). The APC was funded by the Shenzhen Dapeng New District Scientific and Technological Research and Development Fund with grant number (KJYF202001-07), the Guangdong Provincial Special Project in Science and Technology (2021A05240), and the Guangdong Higher Education Institution Innovative Team with grant number (2021KCXTD021).

## Conflict of interest

The authors declare that the research was conducted in the absence of any commercial or financial relationships that could be construed as a potential conflict of interest.

## Publisher's note

All claims expressed in this article are solely those of the authors and do not necessarily represent those of their affiliated organizations, or those of the publisher, the editors and the reviewers. Any product that may be evaluated in this article, or claim that may be made by its manufacturer, is not guaranteed or endorsed by the publisher.
