# Supplementary material for: Hizikia fusiforme functional oil (HFFO) prevents neuroinflammation and memory deficits evoked by lipopolysaccharide/aluminum trichloride in zebrafish
Source: Front Aging Neurosci. 2022 Sep 9;14:941994. doi: 10.3389/fnagi.2022.941994 (PMC9500236; doi:10.3389/fnagi.2022.941994)
Supplement: Supplementary file 1 [file Data_Sheet_1.docx]

Supplementary Material

# Supplementary Tables

**Table S1.** Physicochemical properties of arachidonic acid through online prediction tool of ADMETlab 2.0.

**Table S2.** Physicochemical properties of eicosatrienoic acid through online prediction tool of ADMETlab 2.0.

**Table S3.** Physicochemical properties of palmitic acid through online prediction tool of ADMETlab 2.0.

**Table S4**. Physicochemical properties of tetradecanoic acid through online prediction tool of ADMETlab 2.0.

**Table S5.** Physicochemical properties of phytol through online prediction tool of ADMETlab 2.0.

**Table S6**. Physicochemical properties of 9-hexadecenoic acid through online prediction tool of ADMETlab 2.0

**Table S1** Physicochemical properties of arachidonic acid through online prediction tool of ADMETlab 2.0

| Property | Value | Comment |
| --- | --- | --- |
| Molecular weight | 304.24 g/mol | Optimal: 100~600 g/mol |
| Volume | 358.874 cm^3^/mol | - |
| Density | 0.848 g/cm³ | - |
| Number of hydrogen bond acceptors | 2 | Optimal: 0~12 |
| Number of hydrogen bond donors | 1 | Optimal: 0~7 |
| Number of rotatable bonds | 14 | Optimal: 0~11 |
| Number of rings | 0 | Optimal: 0~6 |
| Number of atoms in the biggest ring | 0 | Optimal: 0~18 |
| Number of heteroatoms | 2 | Optimal: 1~15 |
| Formal charge | 0 | Optimal: -4~4 |
| Number of rigid bonds | 5 | Optimal: 0~30 |
| Flexibility | 2.8 | - |
| Stereo centers | 0 | Optimal: ≤ 2 |
| Topological polar surface area (TPSA) | 37.3 Å² | Optimal: 0~140 Å² |
| Log of the aqueous solubility (LogS) | -2.891 log mol/L | Optimal: -4~0.5 log mol/L |
| Log of the octanol/water partition coefficient (LogP) | 2.505 log mol/L | Optimal: 0~3 |
| LogP at physiological pH 7.4 (LogD) | 3.025 log mol/L | Optimal: 1~3 |

**Table S2** Physicochemical properties of eicosatrienoic acid through online prediction tool of ADMETlab 2.0

| Property | Value | Comment |
| --- | --- | --- |
| Molecular weight | 306.26 g/mol | Optimal: 100~600 g/mol |
| Volume | 361.511 cm^3^/mol | - |
| Density | 0.847 g/cm³ | - |
| Number of hydrogen bond acceptors | 2 | Optimal: 0~12 |
| Number of hydrogen bond donors | 1 | Optimal: 0~7 |
| Number of rotatable bonds | 15 | Optimal: 0~11 |
| Number of rings | 0 | Optimal: 0~6 |
| Number of atoms in the biggest ring | 0 | Optimal: 0~18 |
| Number of heteroatoms | 2 | Optimal: 1~15 |
| Formal charge | 0 | Optimal: -4~4 |
| Number of rigid bonds | 4 | Optimal: 0~30 |
| Flexibility | 3.75 | - |
| Stereo centers | 0 | Optimal: ≤ 2 |
| Topological polar surface area (TPSA) | 37.3Å² | Optimal: 0~140 Å² |
| Log of the aqueous solubility (LogS) | -3.02 log mol/L | Optimal: -4~0.5 log mol/L |
| Log of the octanol/water partition coefficient (LogP) | 4.104 log mol/L | Optimal: 0~3 |
| LogP at physiological pH 7.4 (LogD) | 3.722 log mol/L | Optimal: 1~3 |

**Table S3** Physicochemical properties of palmitic acid through online prediction tool of ADMETlab 2.0

| Property | Value | Comment |
| --- | --- | --- |
| Molecular weight | 256.24 g/mol | Optimal: 100~600 g/mol |
| Volume | 300.236 cm^3^/mol | - |
| Density | 0.853 g/cm³ | - |
| Number of hydrogen bond acceptors | 2 | Optimal: 0~12 |
| Number of hydrogen bond donors | 1 | Optimal: 0~7 |
| Number of rotatable bonds | 14 | Optimal: 0~11 |
| Number of rings | 0 | Optimal: 0~6 |
| Number of atoms in the biggest ring | 0 | Optimal: 0~18 |
| Number of heteroatoms | 2 | Optimal: 1~15 |
| Formal charge | 0 | Optimal: -4~4 |
| Number of rigid bonds | 1 | Optimal: 0~30 |
| Flexibility | 14 | - |
| Stereo centers | 0 | Optimal: ≤ 2 |
| Topological polar surface area (TPSA) | 37.3 Å² | Optimal: 0~140 Å² |
| Log of the aqueous solubility (LogS) | -5.223 log mol/L | Optimal: -4~0.5 log mol/L |
| Log of the octanol/water partition coefficient (LogP) | 6.732 log mol/L | Optimal: 0~3 |
| LogP at physiological pH 7.4 (LogD) | 3.235 log mol/L | Optimal: 1~3 |

**Table S4** Physicochemical properties of tetradecanoic acid through online prediction tool of ADMETlab 2.0

| Property | Value | Comment |
| --- | --- | --- |
| Molecular weight | 228.21 g/mol | Optimal: 100~600 g/mol |
| Volume | 265.64 cm^3^/mol | - |
| Density | 0.859 g/cm³ | - |
| Number of hydrogen bond acceptors | 2 | Optimal: 0~12 |
| Number of hydrogen bond donors | 1 | Optimal: 0~7 |
| Number of rotatable bonds | 12 | Optimal: 0~11 |
| Number of rings | 0 | Optimal: 0~6 |
| Number of atoms in the biggest ring | 0 | Optimal: 0~18 |
| Number of heteroatoms | 2 | Optimal: 1~15 |
| Formal charge | 0 | Optimal: -4~4 |
| Number of rigid bonds | 1 | Optimal: 0~30 |
| Flexibility | 12.0 | - |
| Stereo centers | 0 | Optimal: ≤ 2 |
| Topological polar surface area (TPSA) | 37.3 Å² | Optimal: 0~140 Å² |
| Log of the aqueous solubility (LogS) | -4.378 log mol/L | Optimal: -4~0.5 log mol/L |
| Log of the octanol/water partition coefficient (LogP) | 5.82 log mol/L | Optimal: 0~3 |
| LogP at physiological pH 7.4 (LogD) | 3.023 log mol/L | Optimal: 1~3 |

**Table S5** Physicochemical properties of phytol through online prediction tool of ADMETlab 2.0

| Property | Value | Comment |
| --- | --- | --- |
| Molecular weight | 296.31 g/mol | Optimal: 100~600 g/mol |
| Volume | 360.63 cm^3^/mol | - |
| Density | 0.822 g/cm³ | - |
| Number of hydrogen bond acceptors | 1 | Optimal: 0~12 |
| Number of hydrogen bond donors | 1 | Optimal: 0~7 |
| Number of rotatable bonds | 13 | Optimal: 0~11 |
| Number of rings | 0 | Optimal: 0~6 |
| Number of atoms in the biggest ring | 0 | Optimal: 0~18 |
| Number of heteroatoms | 1 | Optimal: 1~15 |
| Formal charge | 0 | Optimal: -4~4 |
| Number of rigid bonds | 1 | Optimal: 0~30 |
| Flexibility | 13 | - |
| Stereo centers | 2 | Optimal: ≤ 2 |
| Topological polar surface area (TPSA) | 20.23 Å² | Optimal: 0~140 Å² |
| Log of the aqueous solubility (LogS) | -6.311 log mol/L | Optimal: -4~0.5 log mol/L |
| Log of the octanol/water partition coefficient (LogP) | 7.385 log mol/L | Optimal: 0~3 |
| LogP at physiological pH 7.4 (LogD) | 6.093 log mol/L | Optimal: 1~3 |

**Table S6** Physicochemical properties of (Z)-9-Hexadecenoic acid through online prediction tool of ADMETlab 2.0

| Property | Value | Comment |
| --- | --- | --- |
| Molecular weight | 254.22 g/mol | Optimal: 100~600 g/mol |
| Volume | 297.6 cm^3^/mol | - |
| Density | 0.854 g/cm³ | - |
| Number of hydrogen bond acceptors | 2 | Optimal: 0~12 |
| Number of hydrogen bond donors | 1 | Optimal: 0~7 |
| Number of rotatable bonds | 13 | Optimal: 0~11 |
| Number of rings | 0 | Optimal: 0~6 |
| Number of atoms in the biggest ring | 0 | Optimal: 0~18 |
| Number of heteroatoms | 2 | Optimal: 1~15 |
| Formal charge | 0 | Optimal: -4~4 |
| Number of rigid bonds | 2 | Optimal: 0~30 |
| Flexibility | 6.5 | - |
| Stereo centers | 0 | Optimal: ≤ 2 |
| Topological polar surface area (TPSA) | 37.3 Å² | Optimal: 0~140 Å² |
| Log of the aqueous solubility (LogS) | -4.393 log mol/L | Optimal: -4~0.5 log mol/L |
| Log of the octanol/water partition coefficient (LogP) | 5.95 log mol/L | Optimal: 0~3 |
| LogP at physiological pH 7.4 (LogD) | 3.493 log mol/L | Optimal: 1~3 |
